# From SNPs to pathways: integration of functional effect of sequence variations on models of cell signalling pathways

**DOI:** 10.1186/1471-2105-10-S8-S6

**Published:** 2009-08-27

**Authors:** Anna Bauer-Mehren, Laura I Furlong, Michael Rautschka, Ferran Sanz

**Affiliations:** 1 Research Unit on Biomedical Informatics (GRIB), IMIM-Hospital del Mar, Universitat Pompeu Fabra. Barcelona Biomedical Research Park (PRBB) C/Dr. Aiguader, 88, 08003. Barcelona, Spain

## Abstract

**Background:**

Single nucleotide polymorphisms (SNPs) are the most frequent type of sequence variation between individuals, and represent a promising tool for finding genetic determinants of complex diseases and understanding the differences in drug response. In this regard, it is of particular interest to study the effect of non-synonymous SNPs in the context of biological networks such as cell signalling pathways. UniProt provides curated information about the functional and phenotypic effects of sequence variation, including SNPs, as well as on mutations of protein sequences. However, no strategy has been developed to integrate this information with biological networks, with the ultimate goal of studying the impact of the functional effect of SNPs in the structure and dynamics of biological networks.

**Results:**

First, we identified the different challenges posed by the integration of the phenotypic effect of sequence variants and mutations with biological networks. Second, we developed a strategy for the combination of data extracted from public resources, such as UniProt, NCBI dbSNP, Reactome and BioModels. We generated attribute files containing phenotypic and genotypic annotations to the nodes of biological networks, which can be imported into network visualization tools such as Cytoscape. These resources allow the mapping and visualization of mutations and natural variations of human proteins and their phenotypic effect on biological networks (e.g. signalling pathways, protein-protein interaction networks, dynamic models). Finally, an example on the use of the sequence variation data in the dynamics of a network model is presented.

**Conclusion:**

In this paper we present a general strategy for the integration of pathway and sequence variation data for visualization, analysis and modelling purposes, including the study of the functional impact of protein sequence variations on the dynamics of signalling pathways. This is of particular interest when the SNP or mutation is known to be associated to disease. We expect that this approach will help in the study of the functional impact of disease-associated SNPs on the behaviour of cell signalling pathways, which ultimately will lead to a better understanding of the mechanisms underlying complex diseases.

## Background

Single nucleotide polymorphisms (SNPs), among other types of short range sequence variants (see Additional File 1 for definitions of terms), represent the most frequent type of genomic variation between individuals (0.1% of sequence variation in a diploid genome [1]). Moreover, their widespread distribution in the genome and their low mutation rate, have enabled the use of SNPs as genetic markers of phenotypic traits, including diseases. SNPs are currently used in candidate gene association studies, genome wide association studies and in pharmacogenomics studies. Once the SNPs associated with the disease phenotype are identified, the elucidation of the functional effect of predisposing SNP is a key factor for understanding the mechanisms underlying the disease.

Several publications and tools have approached the study of the functional effect of SNPs by assessing their effect on the protein structure or their impact on functional sites at the protein or DNA level [2-6]. All these approaches, although valuable, consider the effect at the single molecule level. It is a well established concept in systems biology that the function of proteins has to be understood through learning how the pathways in which the proteins participate work [7]. In this context, the functional consequences of SNPs are better appreciated if the evaluation is performed at the biological system level, for instance by determining their effect on the dynamics of signalling pathways. In consequence, it is important to consider the effect of SNPs, in particular those having an impact at the protein level (non synonymous SNPs, nsSNPs), in the context of biological networks. Although synonymous SNPs and SNPs located in regions that modulate gene expression (e.g. promoters, introns, splice sites, transcription factor binding sites) can also alter gene or protein function and as a consequence lead to disease [8-11], in this study we focus on nsSNPs as they have a more evident effect on the protein function in the biological processes, and are more prevalent in databases and literature.

The study of the functional consequences of nsSNPs in relation to the molecular basis of diseases requires the integration and aggregation of several pieces of heterogeneous information such as protein sequence and its natural variations, experimental perturbations on protein function, the networks of reactions between proteins, and the phenotypes that are affected by the alterations on the protein function. Several resources collect information about SNPs [12,13] and their association with diseases [2,14] as well as mutations of clinical relevance [15]. The study of protein function is usually assessed by experiments aimed at disrupting the activity of the protein, for instance by means of altering the protein sequence at residues suspected to be critical for the function (e.g. *in vitro *mutagenesis experiments). This information is documented in the biomedical literature, and it has already been recognized that text mining techniques are required to harvest it from free text. Nevertheless, much of this information is already collected in curated databases. One example is the UniProt database [16], which, along with information about protein sequence, structure, and function, records information about the functional effect and the association to disease phenotypes of nsSNPs, referred to as "natural variants" by UniProt. Thus, UniProt provides information about the functional effect of SNPs as well as on the effect of experimental mutation of specific protein residues. This information is recorded as sequence features in each protein entry (see for example , for the entry P00533, in the "Sequence features" section, under "Natural variations" and "Experimental info"). This knowledge is extracted from the biomedical literature by UniProt curators and assigned to the corresponding protein entry [17,18]. Therefore, it represents a reliable source of information about the natural variations of a protein and their associated phenotypes, and on the functional effect of mutations (obtained by experimental mutagenesis of protein residues) on the protein function.

Regarding the participation of proteins in pathways, several databases offer information about models of biological networks such as protein-protein interactions and signalling pathways (for a review on this topic, see [19]). An exemplary resource is Reactome [20], which contains manually curated information about pathways and reactions that involve human proteins. In addition, public repositories of models describing the dynamic behaviour of cellular pathways are also available (see [21] for an example).

With the public availability of resources such as pathway databases and curated datasets on the phenotypic effect of sequence variants, the study of genetic factors that contribute to complex disease phenotypes in the context of the structure and dynamics of biological networks should be feasible. In this regard, there are some reports detailing the integration of SNP data with protein structural data and pathways [22-24]. However, most of them focus on the visualization of nsSNP on the protein structure, and only provide cross references to pathway databases [22,24]. For instance, DataBins [23] is a web service for the retrieval and aggregation of pathway data from KEGG, and sequence databases such as dbSNP [12] with the aim of mapping nsSNPs onto the proteins of a pathway. However, these approaches do not provide any utility for the visualization of nsSNP data on the pathways, not even for analysing the functional effect of the nsSNPs in the pathway context. A different kind of approaches are aimed at using statistical analyses in finding and prioritising metabolic pathways associated with complex diseases based on SNP frequency data (see [25] for an example). However, the functional effects of SNPs have not been incorporated in the analysis. To our knowledge, no strategy has attempted to integrate these sources of information (proteins and their sequence variants such as SNPs, phenotypic effect of SNPs and models of biological networks) with the final goal of assessing the effect of SNPs on the structure and dynamics of biological networks. In this paper we first identify the challenges that have to be faced for performing this integration in an automatic manner. Then, we present a general strategy for the integration of pathway and sequence variation data, towards their use for network visualization and analysis, including the modelling of signalling pathways.

## Results

The goal of this project was to design and implement a general strategy for the integration of pathway and sequence variation data towards their use for network visualization and analysis. In general, in the different models of cellular networks (e.g. signalling pathways, dynamic models, protein-protein interaction networks) the proteins are always represented as nodes, and the edges represent reactions or interactions between proteins. Thus, in practice, the integration involves the mapping of SNPs and mutant residues to the protein nodes of a network and the mapping of their functional effect to the edges of a network (e.g. reactions or relationships between nodes), for their use in the visualization and dynamic analysis of pathways.

In the following sections, we describe and analyse the challenges and approaches for the integration of the phenotypic effect of sequence variations in the context of biological networks, which are:

- Integration of data coming from diverse and heterogeneous sources.

- Visualization of information about sequence variations in the context of biological pathways.

- Incorporation of the effect of the perturbation caused by the sequence variation in dynamic models of the pathways.

### Data integration of the functional effect of SNPs with biological networks

The first step to achieve such an integration is to map SNPs and mutant residues to the protein nodes of a network, and second to map their functional effect to the edges of the network. As described in the Introduction section, UniProt was chosen because it contains manually curated information about nsSNPs and mutant residues of proteins. As described in the Methods section, we identified and extracted human protein entries from UniProt with annotations on natural variation and mutagenesis experiments, which are suitable for integration with biological networks such as protein-protein interaction networks, signalling pathways and dynamic models. In this study we focus on the pathway database Reactome [20] and the dynamic models repository BioModels [21]. The data of these resources are available in standard formats: Reactome reactions and pathways are published in the data exchange format for biological pathway BioPAX [26] (level 2), and dynamic models in the BioModels repository are made available in the SBML standard [27]. As mentioned above, the integration process between the UniProt derived data and the network representations can be considered at two levels. The first level involves the mapping of proteins for which there are natural variation/mutagenesis annotations in UniProt to proteins in biological network models (e.g. signalling pathways, dynamic models, protein-protein interaction networks). This is the simplest task, and was performed by matching the UniProt identifiers from both data sources. In this regard, it is important to note that the different states of a protein such as its level of phosphorylation or its cellular location appear as different entities in a pathway exchange format such as BioPAX and in a model representation such as SBML. However, all the entities that represent different states of a protein are characterized by the same sequence identifiers, e.g. UniProt identifiers. Consequently, the annotations of a given protein were mapped onto all the corresponding instances in Reactome and BioModels, that is, to all the nodes that contain the same UniProt identifier. As a result, data containing the sequence features (natural variations or mutagenesis experiments) extracted from UniProt can be incorporated to visualize, filter and search the biological network, for example using Cytoscape, a software for network visualization [28] (see section "Visualization of SNPs on biological networks" for a complete description).

The second level of data integration involves the incorporation of the effect of the sequence variation in the biological process in which the protein participates. The effect of the sequence variation is expressed in natural language in the Description field of the UniProt files, and comprises one or more phrases. One can be tempted to think that state of the art text mining approaches will easily solve the problem of identification and extraction of the required information in order to map the functional effect onto the biological process represented in the biological model. However, the identification and extraction of the relevant information and its subsequent mapping to the reactions was found to be a non trivial task. An example is presented here in order to illustrate the difficulties that this task implies, and to highlight the challenges that an automatic text mining system should aim to handle. For clarity purposes, the example is analysed from the point of view of a domain expert (e.g. biologist) performing the interpretation of the data and their subsequent integration with pathways.

**Figure 1:**
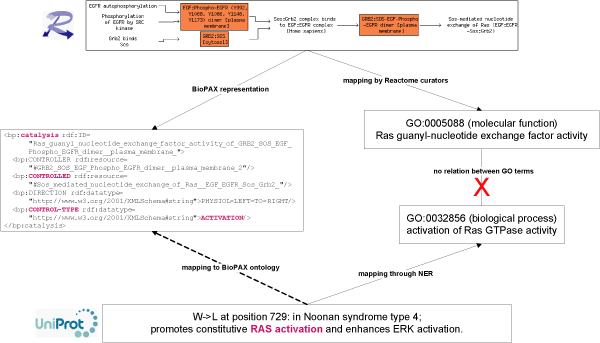
**Mapping SNPs functional effects from textual descriptions to network representations**. The activation of RAS by SOS1 is used as an example. In the upper part of the Figure the Reactome representation of the reaction is depicted. The textual description of the functional effect of the SNP from UniProt is presented in the lower part. In Reactome, the reaction is annotated to the GO term "Ras guanyl-nucleotide exchange factor activity". The type of reaction (catalysis of "control-type activation") is provided by the BioPAX ontology. In UniProt, the W->L mutation at position 729 of SOS1 is described with the text "promotes constitutive RAS activation", which could be mapped to the GO term "activation of Ras GTPase activity" by NER. Reactome and UniProt refer to the reaction through different perspectives impeding a mapping of the UniProt textual description of the sequence variant onto the reaction in Reactome. The direct mapping using the GO annotations is hindered as the two GO terms appear in different GO branches. An alternative would be to use the BioPAX ontology.

The example highlights the natural variant of the SOS1 protein, the W->L change at position 729 of the protein, that has been found to be associated to the Noonan Syndrome type 4, and that is known to promote constitutive RAS activation, therefore enhancing ERK activation (Figure 1). The biologist knows that SOS1 is involved in the EGFR signalling, and he/she is interested in assessing the effect of the natural variation of SOS1, which "promotes constitutive RAS activation and enhances ERK activation", as described in UniProt, in the context of the reactions or interactions in which SOS1 participates. The biologist first identifies the proteins RAS and ERK (ERK1, ERK2), which are mentioned in the textual description of the phenotypic effect of the SOS1 mutation W->L at position 729 of the protein. This information can be used to find the reactions in which the protein participates. At this step, a NER (Named Entity Recognition) system able to perform normalization or disambiguation of the protein symbols to sequence database identifiers, such as UniProt, should be used. This is required for the subsequent mapping of the phenotypic information provided by UniProt to the protein instances in Reactome, which are annotated to UniProt identifiers. Our biologist performs this task manually: he/she queries the Reactome database, using the UniProt identifier of SOS1 [UniProt/Swiss-Prot:Q07889], to retrieve the reactions and pathways in which SOS1 participates. SOS1 is directly involved in the activation of RAS, and this in turn leads to the activation of ERK (Figure 1). In Reactome, the activation of RAS is represented as a chain of reactions that starts with the binding of the EGF ligand to the EGFR and ends with the nucleotide exchange of RAS catalysed by SOS1 (Figure 1). SOS1 is found in the cytoplasm of non stimulated cells in complex with Grb2, and upon binding to activated EGFR receptor complex, SOS1 mediates the nucleotide exchange of RAS, leading to RAS activation. The question here is how to map the effect "promotes constitutive RAS activation" in this chain of reactions. The representation in Reactome depicts the biochemical reactions (e.g. the GTP/GDP exchange of RAS) stimulated by SOS1 that lead to RAS activation. In the textual description of the natural variant effect, the activation of RAS is mentioned, but no detail on the biochemical reaction is given. Thus, the same biological process is referred to in different sources, Reactome and UniProt, through different perspectives, which makes it difficult to identify both representations as the same. A domain expert is able to accomplish this matching on the basis of domain knowledge and inference made from available data (publications, databases). On the other hand, the Reactome reaction is annotated to the Gene Ontology (GO, [29]) term GO:0005088, that represents "Ras guanyl-nucleotide exchange factor activity". Thus, an approach to achieve the mapping in an automatic way would involve finding the GO concepts in the textual description of the SNP. The term RAS activation could be mapped to the concept "activation of Ras GTPase activity" (GO:0032856) by applying NER. Again, these are different concepts describing the same process from different perspectives. Moreover, these two concepts belong to different branches from GO (biological process and molecular funtion), thus hampering the attempt to find a connection between the description of the natural variant and the Reactome reaction using the ontology. However, the connection between the two different perspectives could be achieved using the BioPAX ontology, which describes the reaction ("Sos-mediated nucleotide exchange of Ras (EGF:EGFR-Sos:Grb2)") as a "Catalysis" reaction of the "control-type activation" (see Figure 1 and BioPAX ontology ). In order to be able to use the BioPAX ontology, first the textual description of UniProt has to be mapped to the BioPAX activation reactions and then the entities have to be used to find the specific reaction. For instance the entity RAS could be mapped to the "controlled" entity in the BioPAX representation (Figure 1). In this way, the set of reactions expressed as "Ras activation" in the text could be obtained from the BioPAX representation in Reactome and, eventually, mapped unambiguously.

An additional difficulty appears if the fact that the SNP produces a "constitutive" activation has to be considered as well. But before addressing this issue, the biologist needs to interpret the meaning of "constitutive RAS activation". A possible interpretation of this assertion would be the following: mutated SOS1 does not depend on the binding of the activated EGFR receptor in order to activate RAS, and thus RAS is activated by SOS1 in a constitutive, ligand-independent manner (see [30] for an example). At this stage, an automatic system should deduce that in the presence of the allelic variant W->L of SOS1, there is no requirement for the signal originated by the binding of the EGF to its receptor to activate RAS. To accomplish this, this knowledge should be appropriately represented in an ontology.

In summary, this single example reveals the complexity of the integration process. The steps required to achieve the integration in an automatic way can be expressed as follows:

1. Extraction and mapping of information from natural language description of SNPs and mutations onto reactions or relations in networks. This requires a text mining system able to identify genes/proteins, along with their function and biological process in which they participate.

2. Identification of the entities/relationships in the network and mapping of both representations (text, network). The main difficulties here are the different levels of granularities and different perspectives used in text and pathways to describe the same process.

Solving these challenges will require ontologies and the use of sophisticated text mining tools able to map information extracted from text to information represented in networks. Once the information is represented in a OWL-DL [31] based format, such as Reactome, reasoning could be applied in order to mimic the interpretations performed by a human expert [32-34].

### Mapping of SNPs on biological networks

**Figure 2:**
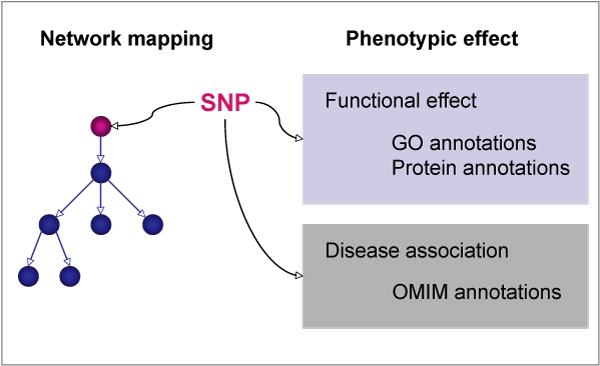
**Schematic view of the integration of SNP phenotypic data with biological networks**.

In order to integrate data about SNPs and protein sequence mutations in biological networks, we developed node attribute files for Cytoscape that allow the visualization of the data in the context of networks. The use of the node attribute files containing protein annotations allows the identification of the nodes in the network that have mutations and/or natural variations. Figure 2 and Table 1 provide information on all the annotations available for each SNP in the attribute files; these annotations can be used to visualize, filter and search the network. As already mentioned, in the pathway representation all different states of a protein appear as different nodes. Hence, we mapped the information about the protein mutation and natural variation of a given protein onto all the corresponding nodes in the pathway. The UniProt identifier was used for this mapping and therefore any pathway, protein-protein interaction network or network model containing UniProt identifiers can be extended with the attribute files. In addition, two distinct visual styles accounting for the network representation formats SBML and BioPAX are provided.

**Table 1:** Node attribute description

**Node attribute name**	**Description**
UniProtId	UniProt identifier
entrezGeneId	Entrez Gene identifier
mutagenesis	List of the mutagenesis information: contains the amino acid exchange, the sequence position and the textual phenotypic description from UniProt
polymorphism	List of the natural variant/polymorphism information: contains the amino acid exchange, the sequence position, the textual phenotypic description from UniProt and if available a MIM id and the textual description of the disease association; if at the same position mutagenesis data is also available, this data is listed as a sub-list of the polymorphism
OMIM	Disease name associated with the natural variant
DbSNP	dbSNP identifier
GObiolProcess	List of GO biological process terms that are associated to the natural variant or mutant
GObiolProcessId	List of GO biological process identifiers that are associated to the natural variant or mutant
GOmolFunction	List of GO molecular function terms that are associated to the natural variant or mutant
GOmolFunctionId	List of GO molecular function identifiers that are associated to the natural variant or mutant
GOcellComponent	List of GO cellular component terms that are associated to the natural variant or mutant
GOcellComponentId	List of GO cellular component identifiers that are associated to the natural variant or mutant
extUniProtIds	List of UniProt identifiers that are associated to the natural variant or mutant
mutPolyFlag	Required for the visual styles 1: only mutagenesis information available 2: only polymorphism information available 3: mutagenesis and polymorphism information available but not at the same position 4: mutagenesis and polymorphism information available at the same position

**Figure 3:**
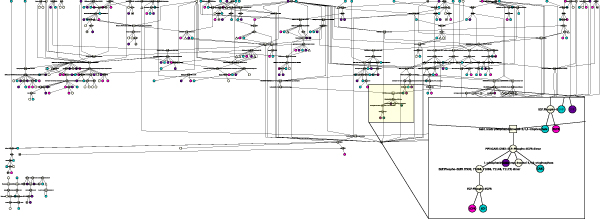
**Cytoscape visualization of the "Signaling by EGFR" pathway in BioPAX format**. The nodes are coloured according to the kind of information they are annotated with (see colour mapping Figure 4).

**Figure 4:**
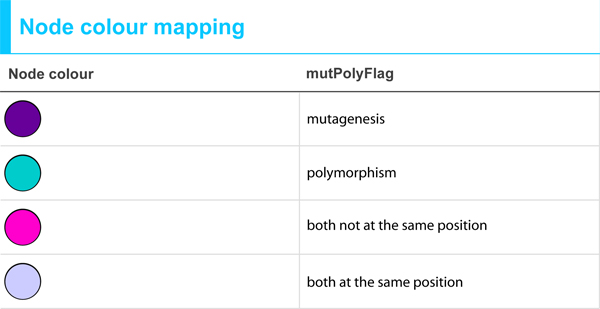
**Node Colour Mapping**. Nodes are assigned to different colours according to the kind of information available. Purple nodes only contain information on mutagenesis experiments; turquoise depicts nodes for which only polymorphism data exists. Some nodes have data for both, mutagenesis and natural variant, either at different sequence positions (pink) or at the same position (light purple).

As an example, Figure 3 shows the complete EGFR signalling pathway in BioPAX format, in which the nodes with annotations on natural variants or mutations are highlighted (see Figure 4 for node colour mapping).

**Figure 5:**
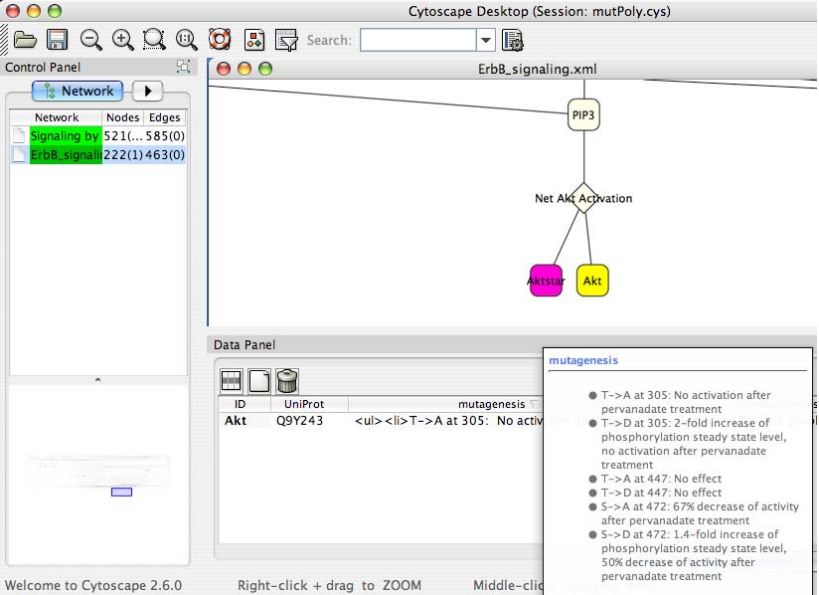
**Cytoscape screenshot depicting part of the "ErbB signalling" (SBML format)**. For the selected node Akt (yellow), the mutagenesis information is shown in the node attribute browser (pop-up window in the lower right part).

**Figure 6:**
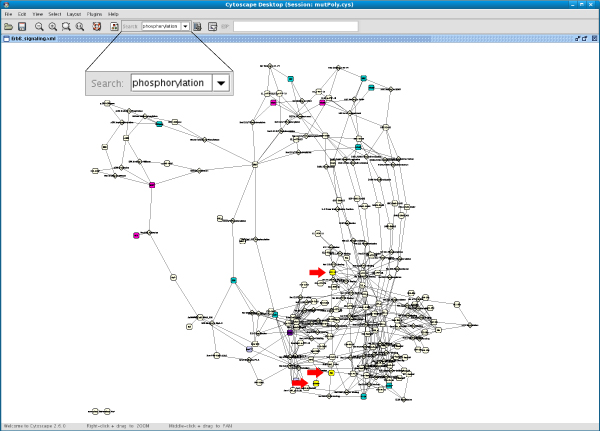
**Cytoscape visualization of "ErbB signalling" (SBML format) after filtering for the GO biological process "phosphorylation"**. All nodes are coloured according to the kind of information they are annotated with (see Figure 4). Nodes that passed the filter are coloured in yellow (see red arrows). They have a variant or mutation that is associated with phosphorylation.

The detailed information about the mutation or natural variant is stored in the node attribute "mutagenesis" or "polymorphism", and can be visualized as a bullet list by moving the mouse over their textual description (see Figure 5). In Figure 5, the Akt activation reaction, which forms part of the ErbB signalling, is depicted in SBML format. The nodes Akt and Aktstar are coloured according to the mutagenesis information available in the attributes file. In the lower right part of the figure, the list of all mutations for the selected node Akt is displayed. For each mutated residue, the position along with the original and changed residue and the phenotypic description accounting for the functional effect of the mutation are provided. Whenever possible, the mutated residue is normalized to dbSNP identifiers. For the natural variants, the position and the original and altered residue, the functional effect and, if available, the disease association including the MIM identifier [15], as well as the dbSNP reference are provided. Moreover, additional annotations (see Table 1) to GO [29] terms and UniProt entries, which were extracted by text mining of the textual description of the mutation, are provided. All annotations can be used for searching or filtering the network on the basis of the functional effect of SNPs. Figure 6 shows the ErbB signalling network (SBML format), in which after applying a filter based on the attribute file, nodes for which the mutation or SNP has an effect on the biological process "phosphorylation" (GO:0013056310) are selected and visualized on the network (coloured in yellow in Figure 6). In this particular example, the amino acid exchange T->D in Akt [UniProt/Swiss-Prot:Q9Y243] at position 305 is associated with a 2-fold increase in phosphorylation.

### Incorporating the effect of perturbation in biological dynamic models

**Figure 7:**
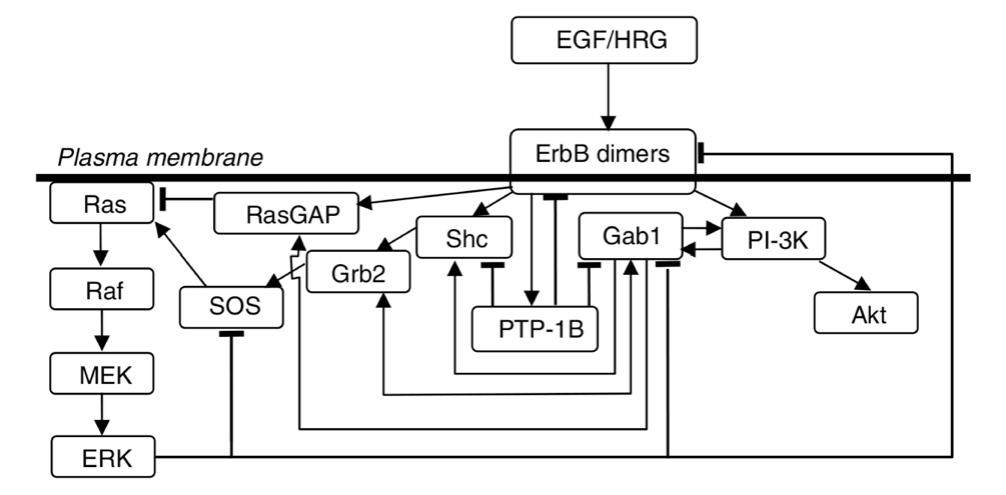
**Simplified schematic representation of the ErbB signalling model**. ErbB receptor ligands (EGF and HRG) activate different ErbB receptor combinations, leading to recruitment of various adapter proteins (Grb2, Shc, and Gab1) and enzymes (PTP1-B, SOS, and RasGAP). These membrane recruitment steps eventually lead to the activation of ERK and Akt. The figure and legends are from the original publication [35].

**Figure 8:**
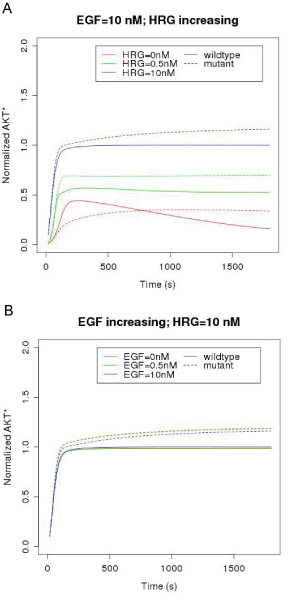
**Dynamic and dose response of the ErbB signalling pathway in the presence of mutated MEK1**. Simulation of Akt activation in response to simulatenous EGF and HGR stimulation. (A) EGF: 10 nM, HGR increasing. (B) HGR 10 n M, EGF increasing. Responses were obtained and normalized as described in [35].

**Figure 9:**
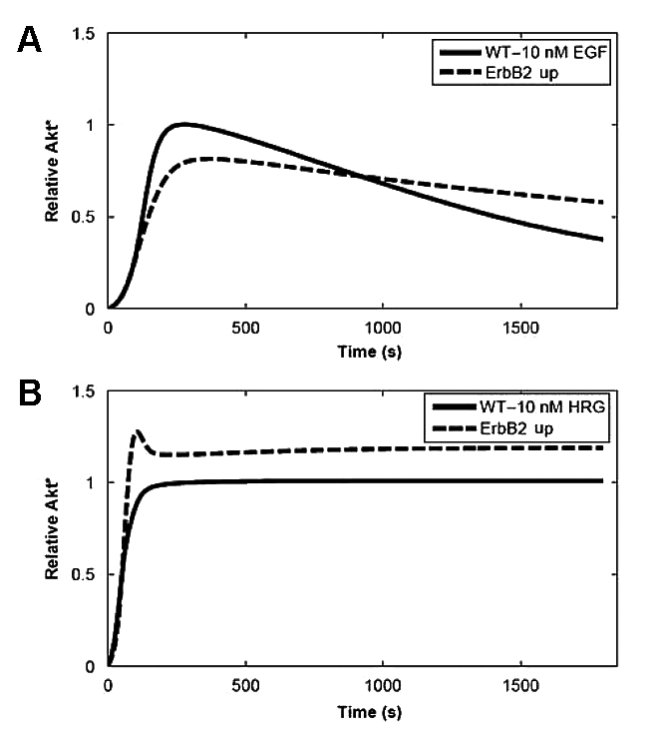
**Effect of 10-fold ErbB2 overexpession**. Simulations of EGF- and HRG-induced Akt activation under wild-type (WT) and ErbB2 overexpression conditions (ErbB2 up). (A) Stimulation with EGFR. (B) Stimulation with HRG. The figure and legends are from the original publication [35].

Once data integration is accomplished, it is desirable to consider this information in the modelling of the behaviour of the network. In particular, it is of interest to determine the effect of different perturbations on the dynamics of a signalling pathway. Here, the perturbations are the functional effects of SNPs or mutations on the activity of the proteins. This task is usually performed by laborious and time-consuming review of the literature. We propose that the integration of the data on perturbations obtained from curated databases such as UniProt with the representation of biological networks can aid in the evaluation of different perturbations on the dynamics of a model. To illustrate how this can be accomplished, the model for the EGFR (or ErbB) signalling network in MCF-7 cells published recently [35] was selected. The ErbB signalling network is composed of multiple extracellular ligands, four trans-membrane receptors (ErbB1 or EGFR, ErbB2 or HER2/NEU, ErbB3, and ErbB4), cytoplasmic adapters, scaffolds, enzymes, and small molecules. Signalling is initiated when a ligand binds to a receptor and causes the receptors to homo- or heterodimerize. This leads to activation of the receptor's tyrosine kinase activity and autophosphorylation of tyrosine residues on receptor cytoplasmic tails. Then, several cytoplasmic adapter, scaffold, and enzymatic proteins can be recruited to the plasma membrane by binding to receptor phosphotyrosines. A complex network of interactions between the activated receptors, recruited proteins, and plasma membrane molecules leads to the activation of multiple downstream effectors, including extracellular-signal-regulated kinase (ERK) and protein kinase B/Akt, which are implicated in the control of proliferation and survival [35,36]. The model is composed of a combination of mechanistic, ordinary differential equations for the representation of the dynamics of the short term response (up to 30 min) of different receptor combinations upon the stimulation with the ligands EGF and HRG. A simplified version of the model is reproduced in Figure 7 from the original publication [35]. In particular, the effect of the S->A mutation at position 218 in MEK1 , which leads to protein inactivation, was evaluated on the dynamics of the network. In order to model this inactivation of MEK1, the species "MEKstar", which represents the activated/phosphorylated MEK1, was modified by changing its concentration to be constantly zero (see Methods). The effect of the MEK1 mutation on the dynamics of the network can be appreciated in Figure 8. The analysis was performed as in the original publication [35], by calculating the amount of activated Akt produced as a response to different combinations of the ligands. Similarly to the dynamics of the response in the wild-type, the response in the network where MEK1 is mutated shows that HRG acts as a dominant ligand. A higher level of active Akt can be observed when the mutant is present in comparison with the wild-type, and a similar response is obtained for all the combinations of EGF/HRG concentrations. Moreover, a remarkable difference was observed when the system was stimulated with EGF 10 nM in the absence of HRG. In this situation, while in the wild-type there is a rapid increase of active Akt peaking at around 4 min, followed by a slower decrease in the signal, in the presence of mutated MEK1, the model predicts a slower rate of formation of active Akt, followed by a mild although sustained increase in the active Akt concentration (no decrease in the concentration up to 30 min was observed). As expected, in the presence of mutated MEK1, the model predicts that active ERK is not produced (data not shown). Based on the ErbB network model (Figure 7), the sustained activation of Akt after stimulation with EGF can be explained by two ERK-dependent inhibitory mechanisms on Akt activation. One is related to the negative feedback loop of ERK on the ErbB receptors, and the other is related to the ERK negative feedback loop on Gab1. In the original publication [35], similar results were obtained when the ERK feedback to the receptors is blocked *in silico*, suggesting that the lack of ERK negative feedback on the receptors leads to a sustained signal. Moreover, a similar response is observed when the ErbB2 receptor is overexpressed (Figure 9). In this situation, the excess of ErbB2 shifts the receptor dimer population towards ErbB1-ErbB2 heterodimers rather than ErbB1-ErbB1 homodimers [35]. Since in the model only ErbB1-ErbB1 homodimers undergo ligand-induced degradation, a more sustained signal is expected (Figure 9). This effect is more evident with EGF since it signals preferably through ErbB1 receptors. In the ErbB2 overexpression model, the slower increase in Akt activation was explained as the result of the increased recruitment of the phosphatase PTP1-B to ErbB1-ErbB2 heterodimers compared to homodimers. As this process is not likely to happen in the MEK1 mutant model, the slower increase in Akt is intriguing. Nevertheless, it is worth to mention that the inactivation of MEK1 is not found to be associated with cancers.

The previous example was only chosen for illustrative purposes, to exemplify the usefulness of incorporating sequence variation data in a modelling exercise.

This approach opens the possibility of evaluating the functional effect of SNPs and mutations on the structure and dynamics of network models.

## Discussion

In this paper we have presented a general strategy for the integration of pathway and sequence variation data, towards their use in network visualization and analysis, as well as in the modelling of signalling pathways. In principle, all the data derived from UniProt could be used for this purpose, provided that the relevant models are available. Several difficulties were found when we tried to combine the data from two structured databases: UniProt and Reactome. Even though the data from these resources is already organized or structured (the entities participating in the interactions are specified) there are a lot of difficulties in the identification of the reactions and nodes in the networks that are affected by the mutation or the SNP. These difficulties go beyond tasks that any current text mining system would be able to handle, since at least NER and relationship extraction tools are required. The difficulties are mainly related with the different perspectives that can be used to refer to the same biological process and how to deal with them to map the different representations to a single concept, and also in the complexity of the processes inherent to the knowledge domain. Similar issues were also discussed in relation to the manual annotation of a corpus describing events in the field of molecular biology [37,38]. In these papers, the authors described the difficulty between mapping events expressed in natural language with reactions represented in pathways.

The intended integration allowing the mapping of the phenotypic effect of SNPs on biological networks (signalling pathways, protein-protein interaction networks, and dynamic models) has evident practical usefulness. The clinical phenotypic effect (e.g. sequence variation associated with colon cancer) and the functional phenotypic effect (e.g. sequence variation produces a decrease of enzymatic activity) can be evaluated in the context of the reactions and processes that are affected by the SNP. This is a very important issue as it provides information about the functional effect of mutations at the cellular level that are relevant in the clinical practice. Disease-associated variants or specific mutations of interest could be evaluated in the context of network models. Moreover, it would be possible to assess the effect of different sequence variations in the same model, an approach particularly relevant to consider the polygenic character of complex diseases. This can have significant consequences for understanding mechanisms of disease and the design of new therapeutical approaches.

## Conclusion

In this paper we have presented a general strategy for the integration of pathway and sequence variation data, towards the use of the integrated information for network visualization and analysis, and for the modelling of signalling pathways. This will aid the modellers in studying the functional impact of protein sequence variations on the model dynamics and proposing relevant experiments. This is of particular interest when the SNP or mutation is known to be associated to disease. We expect that this approach will help in the study of the functional impact of disease-associated SNPs in the behaviour of cell signalling pathways, which ultimately will lead to a better understanding of the mechanisms underlying complex diseases.

## Methods

### Data sources

Mutagenesis and natural variant information was obtained from UniProt/SwissProt (release 57.0 March 2009). The pathway "Signaling by EGFR"  was downloaded in BioPAX format level 2 from Reactome (release 27) (see Additional File 2). The network model of "ErbB signalling" developed by Birthwistle et al. [35] was downloaded in SBML format from BioModels . This model was used for the visualization in Cytoscape [28] and the network modelling. Cytoscape version 2.6.0 supports SBML Level 2 Version 1 (SBML L2 V1). As the model downloaded from BioModels is in SBML L2 V3 format, it had to be modified for visualization in Cytoscape (see Additional File 3). Since the model downloaded from the original publication [35] does not contain a mapping to UniProt identifiers, the mapping between all proteins appearing in the ErbB signalling network and UniProt was obtained from the annotations in the BioModels database and is provided as a mapping file as part of the supplementary materials (see Additional File 4).

### Data integration

**Figure 10:**
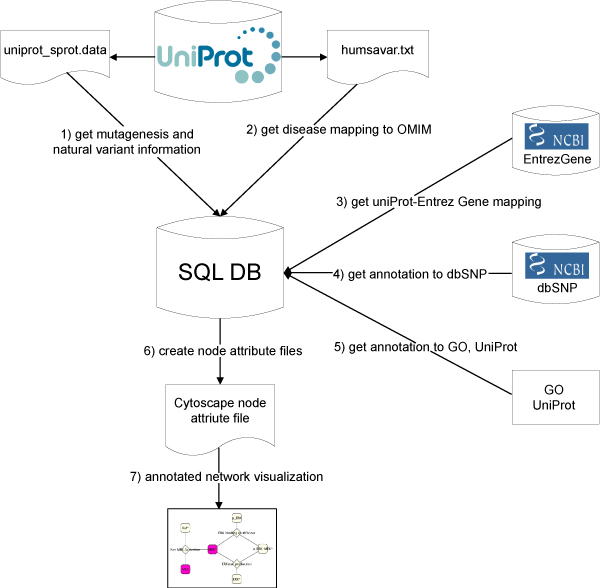
**Workflow of the data integration process**. 1) Data on mutations and sequence variants is gathered from UniProt. 2) The disease association mapping to OMIM is obtained from UniProt, if available. 3) A mapping of UniProt and Entrez Gene ids is created. 4) The annotation to dbSNP, which is in part provided by UniProt is extended. 5) Further annotations to GO ontology are obtained by NER. 6) Cytoscape node attribute files are created from the integrated data that is stored in a SQL database. 7) The annotated network is visualized in Cytoscape.

We extracted the information of mutagenesis experiments and natural variants for all human entries of the manually curated part of UniProt resulting in 11806 entries (Figure 10). For this purpose, we parsed the flat file  of the UniProtKB/Swiss-Prot database using Swissknife [39], an object-oriented Perl library to handle Swiss-Prot entries. UniProt entries contain cross references with several databases, for example to NCBI Gene. However, some entries did not contain the NCBI Gene identifier. Missing mappings to NCBI Gene were obtained from NCBI Gene [40] database whenever possible using mapping obtained from different databases [41]. These mappings were required to complete the annotation of natural variants with dbSNP identifiers (see below). The resulting files are comprised of 54868 natural variants for 11245 UniProt entries, and 7330 mutagenesis experiments for 1766 UniProt entries. For several natural variants and mutants, the description field indicates an association to a disease phenotype. The MIM code for every disease annotation was obtained from the humsavar.txt file , release 57.2 of May 2, 2009), and added to the attribute file as an additional annotation. Annotations to NCBI dbSNP [42] are provided for 24576 natural variants in the original UniProt file. An automatic mapping strategy was applied in order to find additional mappings to dbSNP for natural variants and mutagenesis entries. For this, we obtained the corresponding NCBI Gene identifier for each UniProt entry. This is required in order to have access to dbSNP data, which can be performed through the NCBI Gene identifier. Then we used the position and alleles information from the sequence features annotations to query a local mirror of dbSNP (build 129, NCBI build 36.3) for human genes. We restricted the queries to non-synonymous SNPs, and to different types of SNPs (single base, dips) according to the kind of change described in UniProt. In addition, a correction on the position of the residue was applied to account for differences in counting protein residues raised by considering or not the initial Methionine residue (as described in [43]). Our method is able to provide the correct dbSNP identifier for 94% of the annotations performed by UniProt curators. By this procedure, it was possible to assign dbSNP identifiers to 4505 natural variants and 7 mutagenesis residues that were added to the annotations performed by UniProt curators.

Furthermore, text mining techniques were applied to the text in the Description field in order to identify concepts from controlled vocabularies in the phenotype description of the natural variants and mutations. The EBIMed system [44] was accessed through SOAP web service of Whatizit [45] and applied to the free text. EBIMed contains a NER module for the identification of mentions of GO [29] terms. In addition, it identifies mentions of proteins, which are disambiguated or normalized to UniProt identifiers. These data were extracted as additional annotations on the natural variations and mutations, providing a characterization of the functional effect in terms of GO terms and associated proteins.

All these data were combined for the development of attribute files that can be loaded into Cytoscape allowing the mapping, visualization, filtering and searching of the SNP information in the context of biological pathways.

### Visualization in Cytoscape

For pathway visualization, we used Cytoscape version 2.6.0 [28]. Cytoscape is widely used open-source software for visualization and analysis of networks. In Cytoscape, networks are represented as graphs where the nodes are the entities (e.g. proteins) and the edges their interactions (e.g. reactions). For the visualization of mutagenesis and natural variant information in the context of biological networks, we developed three different node attribute files (for a detailed description of the attributes see Table 1) and two visual style files that can easily be imported into Cytoscape. In detail, we provide separate attribute files for the mutagenesis (see Additional File 5) and the natural variant (polymorphism) data (see Additional File 6) and furthermore one for a combined view of both (see Additional File 7). The two different visual styles account for the two major network representation formats SBML (see Additional File 8) and BioPAX (see Additional File 9) which differ in their node attributes representation. Here, the nodes are coloured according to the kind of information that is available (see Figure 4). Furthermore, we provide a guide explaining how to use the attribute and visual style files within Cytoscape (see Additional File 10), as well as an example Cytoscape session (see Additional File 11). As mentioned above, the mapping of the mutations and natural variants on the pathway requires the existence of UniProt identifiers for the nodes in the pathway. We want to emphasize that for pathways in BioPAX format downloaded from Reactome, there exists a node attribute containing the UniProt identifier. For pathways in SBML format, a mapping of the nodes to UniProt identifiers is provided during curation in BioModels database.

### ErbB signalling network model

We used COPASI (version 4.4) [46] to model the dynamics of ErbB signalling with inactivated MEK. For this purpose, we modified the species "MEKstar", which represents the activated/phosphorylated MEK1, in the SBML file of the original model [35]. We set the species attributes "constant" and "boundaryCondition" to "true" and kept the "initialConcentration" zero. This implies that the concentration of MEKstar is constantly zero. The dynamics of the system and the data normalization were performed as in the original publications. All plots were generated with R [47]. The modified model is available as Additional File 12.

## Abbreviations

SNP: single nucleotide polymorphism; Non-synonymous SNPs: nsSNPs; NER: Named Entity Recognition; SBML Level 2 Version 1: SBML L2 V1; GO: Gene Ontology

## Competing interests

The authors declare that they have no competing interests.

## Authors' contributions

ABM designed and implemented the database, prepared all data files, developed the files for the visualization and performed the modelling. MR participated in the implementation of the database and in preparation of data files. LIF led the design and coordination of the study, participated in the implementation, evaluation and analysis of the results, and prepared the first draft of the manuscript. FS participated in the design and coordination of the work. All authors contributed to the manuscript, and read and approved its final version.

## Supplementary Material

Additional file 1 Glossary and identifiers and names of proteins used in the study. Click here for file

Additional file 2 BioPAX file of Signaling by EGFR pathway from Reactome. Click here for file

Additional file 3 SBML file containing the original model of ErbB signaling by [35], slightly modified to be imported to Cytoscape. Click here for file

Additional file 4 Cytoscape node attribute file containing the mapping of the nodes in ErbB_signaling (SBML) to UniProt identifiers. Click here for file

Additional file 5 Cytoscape node attribute file only containing information about mutations. Click here for file

Additional file 6 Cytoscape node attribute file containing only information about polymorphism. Click here for file

Additional file 7 Cytoscape node attribute file containing information about mutagenesis and polymorphisms. Click here for file

Additional file 8 Visual style file for SBML format. Click here for file

Additional file 9 Visual style file for BioPAX format. Click here for file

Additional file 10 Guide to map SNP data onto biological networks (brief tutorial on how to use the files). Click here for file

Additional file 11 Example Cytoscape session where the EGFR signalling pathway from Reactome in BioPAX format, as well as the network model in SBML format are imported and visualized with the mutagenesis and polymorphism data Click here for file

Additional file 12 SBML model of ErbB signalling, in which MEKstar is inactivated. Click here for file
