# Supplementary material for: From SNPs to pathways: integration of functional effect of sequence variations on models of cell signalling pathways
Source: BMC Bioinformatics. 2009 Aug 27;10(Suppl 8):S6. doi: 10.1186/1471-2105-10-S8-S6 (PMC2745588; doi:10.1186/1471-2105-10-S8-S6)
Supplement: Additional file 1 — Glossary and identifiers and names of proteins used in the study. [file 1471-2105-10-S8-S6-S1.pdf]

From SNPs to pathways: Integration of functional effect of sequence variations on models of cell signalling pathways

Anna Bauer-Mehren, Laura I Furlong[§], Michael Rautschka, Ferran Sanz

Glossary

Variation: any kind of short range sequence variation in the nucleotide sequence of the genome. Among these we can mention SNPs and insertion-deletion variants of a small number of nucleotides, Alu sequences and other types of variations collected in dbSNP database .

SNPs: single nucleotide polymorphisms or SNPs are DNA sequence variations in which a single nucleotide (A, G, C or T) is altered. SNPs are also referred as polymorphisms, natural variants, or common variants because they have a minor allele frequency in the population of at least 1 %. In contrast, rare variants have a minor allele frequency of less than 1 % . SNPs can either be neutral or have an effect on gene or protein function.

Mutation: term used to refer to rare variants (minor allele frequency less than 1 % in the population). These variants often cause disease and segregate within families, and affect conserved residues in the protein sequence. Also used to refer to modified residues in a sequence after the experimental procedure of mutagenesis.

Mutagenesis: experimental procedure aimed at altering the protein sequence at residues suspected to be critical for the function of the protein.

Identifiers and names of proteins used in the study

Protein name (as used in this study)	UniProt id	Protein name according to UniProt
SOS1	Q07889	Son of sevenless homolog 1
RAS (generic term for HRas, KRas, NRas)	P01112	GTPase Hras
	P01116	GTPase Kras
	P01111	GTPase Nras
ERK (generic term for ERK1, ERK2)	P27361	Mitogen-activated protein kinase 3 (ERK1)
	P28382	Mitogen-activated protein kinase 1 (ERK2)
EGFR or ErbB1	P00533	Epidermal growth factor receptor
Grb2	P62993	Growth factor receptor-bound protein 2
EGF	P01133	Pro-epidermal growth factor
MEK (generic term for MEK1, MEK2)	Q02750	Dual specificity mitogen-activated protein kinase 1 (MEK1)
	P36507	Dual specificity mitgen-activated protein kinase 2 (MEK2)
MEKstar (activated MEK1)	Q02750	Dual specificity mitogen-activated protein kinase 1 (MEK1)
RAF	P04049	RAF proto-oncogene serine/threonine-protein kinase
Akt	Q9Y243	RAC-gamma serine/threonine-protein kinase
Aktstar (activated Akt)	Q9Y243	RAC-gamma serine/threonine-protein kinase
ErbB2 or HER2/NEU	P04626	Receptor tyrosine-protein kinase

		erbB-2
ErbB3	P21860	Receptor tyrosine-protein kinase erbB-3
ErbB4	Q15303	Receptor tyrosine-protein kinase erbB-4
HRG	Q02297	Pro-neuregulin-1, membrane-bound isoform (heregulin)
Gab1	Q13480	GRB2-associated-binding protein 1
PTP1-B	P18031	Tyrosine-protein phosphatase non-receptor type 1
