# Supplementary material for: From SNPs to pathways: integration of functional effect of sequence variations on models of cell signalling pathways
Source: BMC Bioinformatics. 2009 Aug 27;10(Suppl 8):S6. doi: 10.1186/1471-2105-10-S8-S6 (PMC2745588; doi:10.1186/1471-2105-10-S8-S6)
Supplement: Additional file 10 — Guide to map SNP data onto biological networks (brief tutorial on how to use the files). [file 1471-2105-10-S8-S6-S10.pdf]

From SNPs to pathways: Integration of functional effect of sequence variations on models of cell signalling pathways

Anna Bauer-Mehren, Laura I Furlong[§], Michael Rautschka, Ferran Sanz

Research Unit on Biomedical Informatics (GRIB), IMIM-Hospital del Mar, Universitat Pompeu Fabra. C/Dr. Aiguader, 88, 08003. Barcelona, Spain

[§]Corresponding author

Guide to map SNP data onto biological networks

A: Import networks..2

B: Import node attributes..3

C: Import visual style...4

A: Import networks

A.1: Import network from file

1. File->Import->Network (multiple file types)…
2. Select network file
    a. EGFR_signaling.owl (BioPAX format)
    b. ErbB_signaling.xml (SBML format)

    [UniProt id attribute does not exist, for mapping SNP data onto this pathway, please import first the UniProt_mapping_ErbB_signaling.attr as described in **B**]

    c. any other pathway
3. Import
4. Optional: Change network layout

A.2: Import network from webservice

Please check:

http://www.cytoscape.org/cgi-bin/moin.cgi/Cytoscape_User_Manual/ImportingNetworksFromWebServices

B: Import node attributes

1. Make sure that there exists a node attribute containing the UniProt identifier [if you have imported ErbB_signaling.xml, you need to import UniProt_mapping_ErbB_signaling.attr first]
2. File->Import->Attribute from Table (Text/MS Excel)…
3. Select attribute file (see supplementary material)
    a. Mutagenesis data only (mutagenesis.attr)
    b. Polymorphism data only (polymorphism.attr)
    c. Combination of both (mutPoly.attr)
4. Enable options
    a. In the “Advanced” section: Show Mapping Options
    b. In the “Advanced” section: Show Text File Import Options
    c. In the “Text File Import Options” section: Transfer first line as attribute names
5. In the “Advanced” section: Select Key Column in Annotation File: Select as Primary Key “uniProtId”
6. Select as Key Attribute for Network the attribute that contains the UniProt id
7. Import
8. Enable the attributes of interest (for detailed description see Table 2) in the node attribute browser in the data panel

C: Import visual style

1. Make sure one of the attribute files is imported
2. File->Import->Vizmap Property File…
3. Select visual style of interest (see supplementary material)
    a. mutPoly_BioPAX.props for pathways in BioPAX format
    b. mutPoly_SBML.props for pathways in SBML format
4. Select the appropriate visual style in the VizMapper
